# Quality of Life and Mental Health Benefits of Public Participation in Forest Conservation Activities in Urban Areas

**DOI:** 10.3390/ijerph19159768

**Published:** 2022-08-08

**Authors:** Dawou Joung, Bum-Jin Park, Shinkwang Kang

**Affiliations:** 1 Institute of Agricultural Science, Chungnam National University, Deajeon 34134, Korea; 2 Department of Environment and Forest Resources, Chungnam National University, Deajeon 34134, Korea; 3 Department of Thoracic and Cardiovascular Surgery, Chungnam National University Hospital, School of Medicine, Chungnam National University, Deajeon 35015, Korea

**Keywords:** green gym, musculoskeletal system, physical activity, psychological effects, urban forest

## Abstract

The purpose of this study is to investigate the effect of forest conservation activities on the physical and psychological wellbeing of participants. The experiment was conducted in a forest near an urban area and involved 61 participants (average age: 22.5 ± 1.8). The participants selected one of three activities (pruning, stacking cut branches, and removing vines) in the forest conservation program. The effects of these activities on the musculoskeletal system were assessed using the Ovako Working Posture Assessment System (OWAS); the physical intensity of the activities was evaluated using heart rate data. The psychological evaluation measurement indexes used the Positive and Negative Affect Schedule, Rosenberg Self-Esteem scale, World Health Organization Quality of Life assessment instrument, and the Perceived Restorativeness Scale. As a result of the OWAS assessment, forest conservation activities were found to be action categories 1 and 2, which were less burdensome to the musculoskeletal system. All forestry activities were found to be light levels of physical intensity. Psychological evaluation of the participants revealed that positive emotions such as self-esteem, quality of life, and perceived restorativeness increased significantly, whereas negative emotions decreased significantly. This forest conservation program, that involved low-intensity activities which were less burdensome to the musculoskeletal system, had positive physical and psychological effects on the local residents who participated.

## 1. Introduction

Since the advent of mankind, humans have primarily lived in natural habitats and have evolved by adapting to the natural environment. However, industrialization and economic growth after the 1950s have resulted in rapid urbanization [1], and the urbanization rate, which was only 29.6% in 1950, had reached 55.3% in 2018 [2]. This acceleration of industrialization and urbanization has resulted in a decrease in forested areas and has aggravated the atmospheric environment resulting in problems [3,4] such as fine dust [5] and heat waves [6], which negatively affect mental health and cause various physical diseases [7,8,9].

In contrast, green spaces have been reported to not only improve the quality of the atmospheric environment [10,11,12] but also offer opportunities of physical activity to modern people [13,14,15], which have positive effects on individual’s physiological and psychological stability [16,17,18]. It is essential to restore and preserve green spaces, for society to continue to reap the benefits. To effectively sustain green spaces in urban areas, local residents can be involved in environmentally-focused programs through civic ecology, civic participation, and community-based management [19]. Such public-participation projects offer cultural ecosystem services, including environmental education, and can generate positive, self-reinforcing feedback [20,21]. In the case of the United Kingdom, Green Gyms are being actively promoted through green space creation and conservation programs with citizen participation. Since its initiation in 1997, 58 regional networks have been established and are currently operating across the country. A green gym is a work activity based on physical activities to provide a green space to the local community and aid environmental conservation through tree planting, pruning, and forest road maintenance; it further aims to improve the physical and mental health of the participants [22,23].

As human coexistence with the environment is gaining importance, green space management projects with citizen participation, such as green gyms, can be an alternative. Research on the health benefits of forest conservation activities (FCAs) and their effect on the quality of life are necessary. Therefore, this study was conducted to investigate the physical and psychological effects of FCAs on participants.

## 2. Materials and Methods

### 2.1. Participants

In this study, physically-healthy young adults in their 20s were recruited as participants via advertisements posted on campus bulletin boards, social networking services, and websites. Participants with a history of smoking, drinking, and drug use were excluded. A total of 61 participants (35 males; 26 females; average age, 22.5 ± 1.8 years) voluntarily participated in the study. The sample size requirement was calculated using the G*Power software version 3.1.9.2 (University of Kiel, Kiel, Germany) [24]. Based on these calculations, a minimum of 57 participants were required to detect statistically significant differences in the two-tailed test with an effect size of 0.5, significance level of 0.05, and power of 0.95; thus, the sample size of 61 participants used in this study was sufficient to test statistical significance. The participants were informed of the study contents prior to participation, and they proceeded only after being thoroughly familiarized with the study method. The study was conducted according to the guidelines of the Declaration of Helsinki, and all experimental procedures were performed following a review by the Bioethics Review Committee of Chungnam National University (IRB No. 201709-SB-023-01).

### 2.2. Research Sites

**Figure 1:**
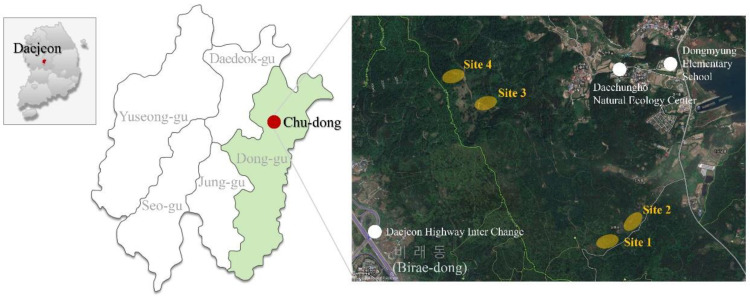
The location of the study area and experimental sites.

The research area of this study was an urban forest located in Chu-dong, Dong-gu, Daejeon, Republic of Korea, and the experiments were conducted in a total of four locations (Figure 1, Table 1). Site 1 was a coniferous forest composed of *Metasequoia glyptostroboides* with a 10° ground slope; the trees had an average height of 15 m and an average diameter at breast height (DBH) of 16 cm. Site 2 was a coniferous forest composed of *Picea abies* (L.) H. Karst., with a ground slope of 10°, average height of 20 m, and average DBH of 14 cm. Activities conducted in sites 1 and 2 included pruning using a hand saw and stacking the pruned branches. Sites 3 and 4 were non-stocked forest land composed of less than 30% standing trees with a ground slope of 15°; the activity conducted at these sites was the removal of vines using pruning shears.

### 2.3. Forest Conservation Activity Program

#### 2.3.1. Warm-Up Exercise

Warm-up exercises were performed to prevent the participants from experiencing physical problems while working, due to sudden physical activity. These exercises also fostered camaraderie among the participants.

#### 2.3.2. Expert Demonstration and Safety Training

The operation of the equipment used in the forest conservation activity (FCA) program was explained through a demonstration by an expert, for participants who were unfamiliar with their use. Additionally, participants with educated on general safety guidelines to prevent accidents during the activity. A target workload was not suggested in order to prevent participants from overworking to achieve the target. Additionally, participants were instructed to rest if they felt fatigued during the activity.

#### 2.3.3. First FCA Session

FCA allows the cultivation and preservation of forests and includes activities such as tree planting, mowing, pruning, logging, and forest road maintenance. For this study, only pruning, stacking pruned branches, and vine-removal were conducted; each participant had the choice of performing an activity according to their physical strength and interest. Meanwhile, adults are advised to engage in physical activities for at least 10 min [25] and accumulate 30 min or more of moderate-intensity physical activity on most, if not all, days of the week [26]. Therefore, the FCA was set at 20 min per session. To meet the minimum time recommended for daily physical activity, two sessions with a total accumulated time of 40 min were conducted. Furthermore, to prevent the participants from perceiving the forest conservation activity as labor, we decided to divide the total accumulated time of 40 min into two short 20-min sessions.

#### 2.3.4. Break Time

There was a break between the 1st and 2nd FCA where the participants and manager gathered for snacks and to share experiences, fostering a community spirit among the participants regardless of their age and gender.

#### 2.3.5. Second FCA Session

The second FCA session was also conducted for a period of 20 min, similar to the 1st FCA.

#### 2.3.6. Cool-Down and Finish

After finishing all FCA, a cool-down was performed. In contrast to the warm-up, a cool-down session was conducted to allow the pulse and respiratory rate to gradually normalize. The equipment was organized, participants shared comments and feedback, and the program was concluded.

### 2.4. Procedures

**Figure 2:**
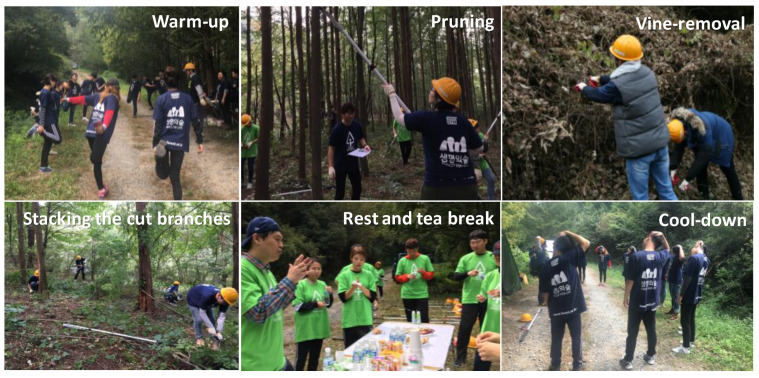
FCA program.

**Figure 3:**
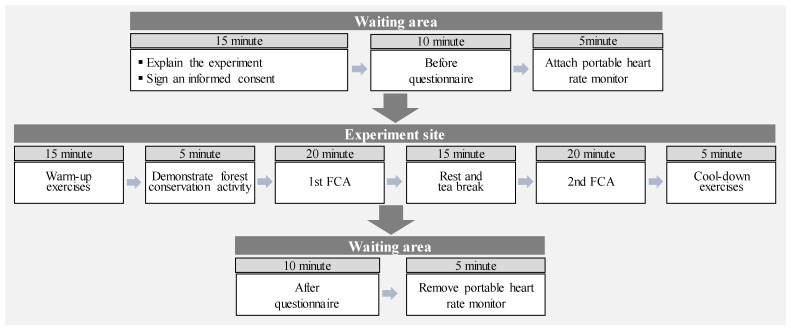
Experimental procedure flow chart.

This study was conducted between September 2017 and November 2017; the experimental procedures are shown in Figure 2 and Figure 3. First, while the participants were waiting in the waiting area, they were provided with explanations with regard to the purpose, outline, and research methods of this study. All study participants signed a written consent to participate in the study at this time. All participants responded to a total of four pre-questionnaires, and a portable heart rate monitor was attached to the participants’ bodies in order to measure heart rate during the FCA. Subsequently the participants participated in the FCA program, which consisted of the following: ① Warm-up exercise, ② Expert demonstration and safety training, ③ 1st FCA, ④ Break, ⑤ 2nd FCA, and ⑥ Cool-down. The program lasted for a total of 80 min. After the FCA program concluded, the participants responded to four post-questionnaires same to the pre-questionnaires, in the waiting area, and the portable heart rate monitors attached to the participants’ bodies were removed.

### 2.5. Physical Activity Measurement Indicators

#### 2.5.1. Musculoskeletal System Load

The Ovako Working Posture Assessment System (OWAS), developed by the Finnish steel company Ovako and the Finnish Institute of Occupational Health in the 1970s, was used to evaluate the degree of musculoskeletal burden. OWAS allows the representative assessment of the degree of musculoskeletal burden through observations of the working posture [27] at regular time intervals analyzed as per the OWAS classification system.

**Figure 4:**
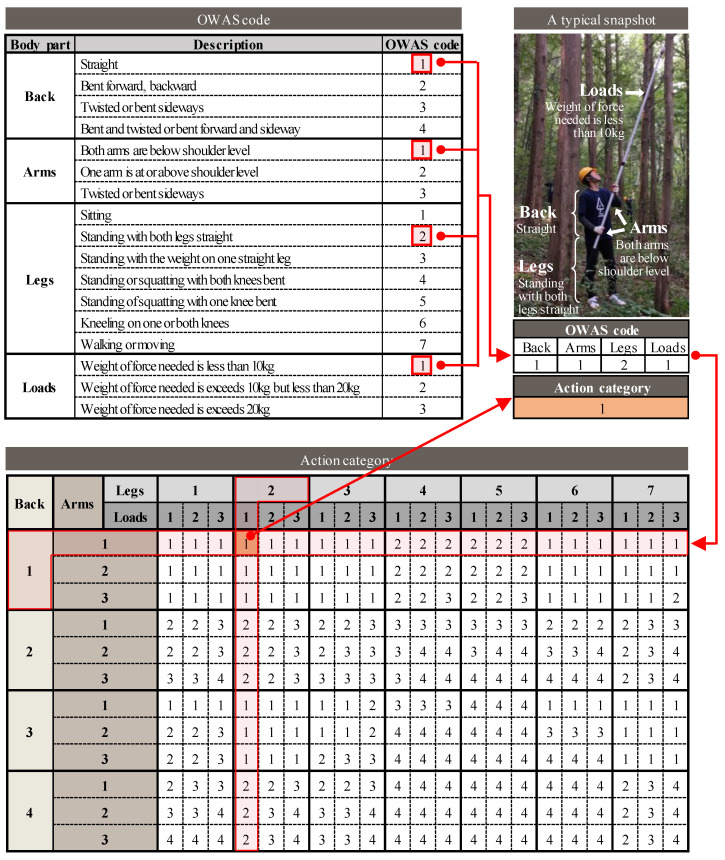
The concept behind Ovako Working posture Assessment System.

The work activities of five participants, who agreed to be filmed, were recorded at regular intervals, to evaluate their postures. Image data was extracted every 30 s, and the working posture was classified according to the OWAS working posture classification system. This system analyzes the working posture as a work code, which evaluates the load on three body parts: the waist, upper extremities (arms), and lower extremities (legs), and tools (Figure 4). Additionally, the level of musculoskeletal burden can be evaluated by combining the work codes for each body part and load, divided into four action categories (AC), according to their effect on the musculoskeletal system (Table 2).

#### 2.5.2. Physical Activity Intensity

To measure the intensity of physical activity while participants were engaged in FCAs, the percentage of heart rate reserve (%HRR) was used, which allows the correction of differences in individual work ability by showing a relative value of exercise intensity.

The heart rate reserve (HRR) is calculated by subtracting resting heart rate (HR_r_) from the maximum heart rate (HR_max_). The %HRR is the percentage calculated using the following equation:

HR_w_ − HR_r_/HRR × 100

where HR_r_ is the resting heart rate and HR_w_ is the working heart rate.

Table 3 shows the classification criteria for physical activity intensity according to %HRR [28]. Meanwhile, HR_max_ refers to the maximum heart rate an individual can achieve in a minute. In this study, the commonly used maximum heart rate prediction formula (HR_max_ = 220 − age) was applied [29].

### 2.6. Mental Health Measurement Indicators

#### 2.6.1. Quality of Life

In this study, the Korean version of the World Health Organization Quality of Life framework (WHOQOL-BREF), developed by the WHO to measure the quality of life, was used. The Korean version of the WHOQOL-BREF consists of 26 questions. Excluding two questions related to the overall quality of life and health status, the remaining 24 questions consist of the following domains: ① Physical health (7 questions), ② Psychological (6 questions), ③ Social relationships (3 questions), and ④ Environment (8 questions). Each domain is evaluated using a 5-point Likert scale, and a higher score represents a higher quality of life. The score for each sub-domain was calculated by converting the scores for each domain to a 100-point scale, according to the calculation method suggested under standard guidelines [30]. In this study, Cronbach’s α, a reliability coefficient, was 0.927 for WHOQOL-BREF; 0.797 for the physical domain; 0.835, psychological health domain; 0.683, social relationship domain; and 0.844, environmental domain.

#### 2.6.2. Rosenberg Self-Esteem Scale (RES)

In this study, the RES was used to measure self-esteem [31]. It is the most frequently used scale among existing scales for self-esteem and evaluates the degree to which one perceives oneself positively and as valuable. The RES consists of ten items evaluated using a 4-point Likert scale. The higher the total score of all items, the higher is the assessed level of self-esteem. In this study, the Korean version was used [32], and the reliability coefficient Cronbach’s α, which represented internal consistency, was 0.642.

#### 2.6.3. Positive and Negative Affect Schedule (PANAS)

PANAS is a scale developed to measure positive and negative emotions and has been proven to be reliable and valid regardless of age and nationality, in studies that involve diverse groups. In this study, we used the PANAS scale developed by Watson et al. (1988) [33] and validated by Park and Lee (2016) [34]. It consists of 20 items, with ten adjectives measuring positive and negative emotions each. Each item evaluates emotions using a 5-point Likert scale. In this study, the reliability coefficient Cronbach’s α was 0.781; as a subset, that for positive emotions was 0.830 and negative emotions, 0.870.

#### 2.6.4. Perceived Restorativeness Scale (PRS)

The PRS is a measure of how subjectively one perceives the four characteristics of a recovery environment: Being-away, Extent, Fascination, and Compatibility. In this study, we used the PRS developed by Hartig et al. (1997) [35] and translated by Lee and Hyun (2003) [36]. The Korean version of the PRS consists of 26 questions, and each is evaluated using a 5-point Likert scale. The total score assessed as the score of the recovery environment of the relevant environment. The reliability coefficient Cronbach’s α of the Korean version of the PRS used in this study was 0.969.

### 2.7. Statistical Analysis

All statistical analyses were performed using the Statistical Package for Social Sciences software version 24.0 (IBM Corp., Armonk, NY, USA). The Wilcoxon signed-rank test was used to compare changes in the quality of life and mental health before and after participating in the FCA. This study used a two-sided test, and *p* < 0.05 indicated statistical significance.

## 3. Results

### 3.1. Musculoskeletal System Load

The OWAS analysis was performed to evaluate the effect of FCA on musculoskeletal loads (Table 4). Results showed that all FCAs had the highest proportion of activities rated as work level 1, which did not cause any particular harm to the musculoskeletal system. In the average action category, pruning was 1.69 ± 0.11, stacking cut branches was 1.85 ± 0.14, and vine-removal was 2.21 ± 0.12. Accordingly, the average working posture level of all FCAs conducted in this study was either level 1 or level 2, indicating that FCA had a low burden on the musculoskeletal system.

### 3.2. Physical Activity Intensity (PAI)

**Figure 5:**
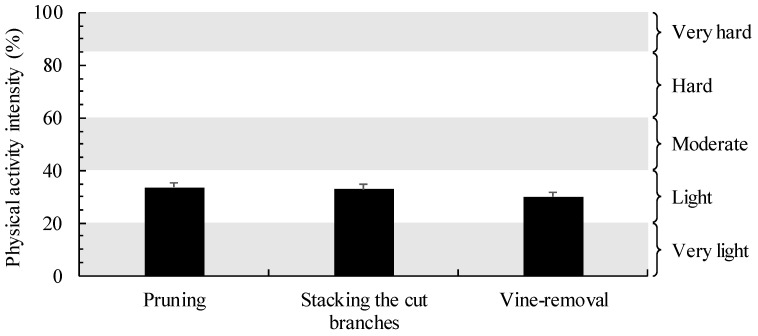
Comparison of the PAI of each FCA. Data are presented as mean ± standard error.

To calculate the PAI of FCA, the maximum heart rate, resting heart rate, and working heart rate of each detailed activity of the FCA were investigated (Table 5, Figure 5).

The calculated maximum heart rates associated with each activity were as follows: pruning, 197.8 ± 0.2 bpm; stacking cut branches, 197.7 ± 0.2 bpm; and vine-removal, 196.6 ± 0.3 bpm. The resting heart rates associated with each activity were as follows: pruning, 78.9 ± 1.5 bpm; stacking cut branches, 78.8 ±1.7 bpm; and vine-removal, 81.9 ± 1.9 bpm. The working heart rate while pruning was 119.4 ± 1.6 bpm; stacking cut branches, 118.6 ±1.9 bpm; and vine-removal, 116.2 ± 2.6 bpm.

Based on the PAI evaluation, pruning was found to have the highest work intensity at 33.6 ± 1.6%, followed by stacking cut branches (32.8 ± 1.8%), and vine-removal (29.9 ± 1.9%). These results reflect that all FCAs conducted in this study had light PAI.

### 3.3. Quality of Life

**Figure 6:**
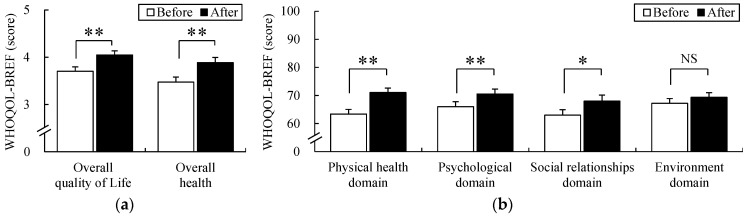
Changes in scores of WHOQOL-BREF before and after the FCA (**a**) The individual’s overall perception of quality of life and health. (**b**) The four domains (physical health, psychological health, social relationships, environment health) of WHOQOL-BREF. Data are presented as mean ± standard error (*n* = 61). Significance was verified by the Wilcoxon signed-rank test; NS = not significant, * *p* < 0.05, ** *p* < 0.01.

The assessed quality of life before and after participating in FCA was investigated using the WHOQOL-BREF (Figure 6). The average overall quality of life score before participating in FCA was 3.70 ± 0.09 and that after participation was 4.05 ± 0.09. This reflected a significant increase of 9.46% as compared prior to participation (*p* < 0.01). Additionally, the average score of overall health before participating in FCA was 3.48 ± 0.11 and that after participation was 3.89 ± 0.11, which indicated a significant increase of 11.78% as compared prior to participation (*p* < 0.01). The four domains of WHOQOL-BREF were evaluated. The average scores in the physical health domain before and after participating in FCA were 63.43 ± 1.63 and 71.03 ± 1.61, respectively, which was a significant increase of 11.98% as compared prior to participation (*p* < 0.01). The average score in the psychological health domain before participating in FCA was 65.98 ± 1.77 and that after participation was 70.54 ± 1.75, which was a significant increase of 6.91% compared to before participation (*p* < 0.01). In the case of the social relationships domain, the average score before participating in FCA was 62.98 ± 1.97 and after participation, 67.97 ± 2.18; this showed a significant increase of 7.92% compared to before participation (*p* < 0.01). The average score of the environmental health domain before participating in FCA was 67.18 ± 1.75, and the average score after participation was 69.36 ± 1.63; although it increased from before participation, it was not statistically significant.

### 3.4. Rosenberg Self-Esteem Scale

**Figure 7:**
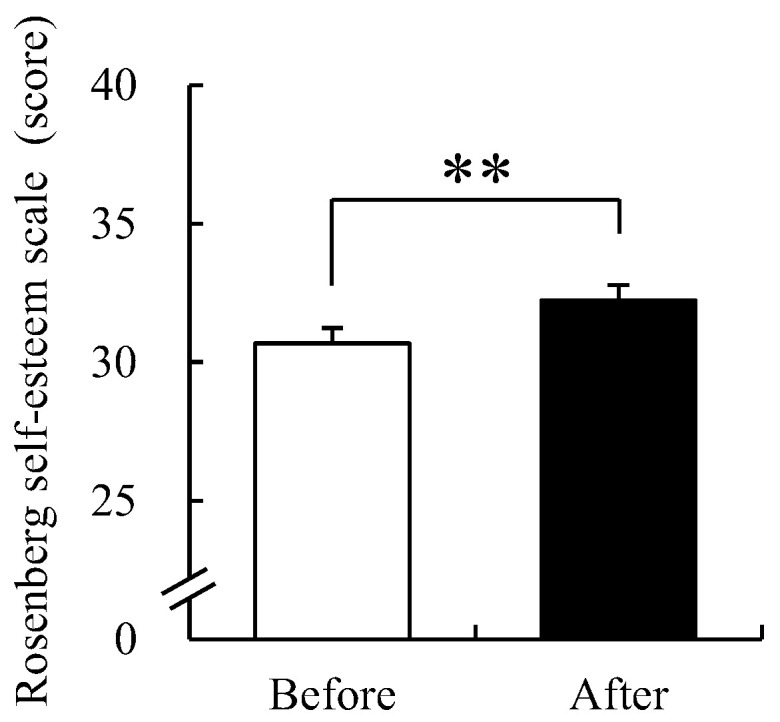
Changes in RES scores before and after the FCA. Data are presented as mean ± standard error (*n* = 61). Significance was verified by the Wilcoxon signed-rank test; ** *p* < 0.01.

Self-esteem before and after participating in FCA was investigated using the RES (Figure 7). The average self-esteem score before participating in FCA was 31.44 ± 0.43 points and that after participation was 33.17 ± 0.42. This was a significant increase of 5.50% as compared prior to participation (*p* < 0.01). These results reflect that FCA was effective in enhancing self-esteem.

### 3.5. Positive and Negative Affect Schedule

**Figure 8:**
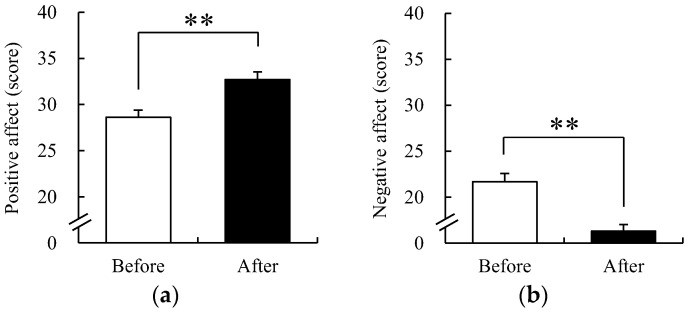
Changes in scores of positive affect and negative affect schedule (PANAS) before and after the FCA. (**a**) Positive affect of PANAS; (**b**) Negative affect of PANAS. Data are presented as mean ± standard error (*n* = 61). Significance was verified by the Wilcoxon signed-rank test; ** *p* < 0.01.

Positive and negative emotions were investigated before and after participation in FCA using PANAS (Figure 8). As a result, the average score of positive emotion prior to participation in FCA was 30.16 ± 0.66 and that after participation was 35.17 ± 0.75. This was a significant increase of 16.61% as compared prior to participation (*p* < 0.01). The average negative emotion score before participating in FCA was 21.07 ± 0.74; the average negative emotion score after participating in FCA was 15.33 ± 0.51. This was a significant reduction of 27.24% as compared prior to participation (*p* < 0.01). These results suggest that FCA was effective in enhancing positive emotions and reducing negative emotions.

### 3.6. Perceived Restorativeness Scale

**Figure 9:**
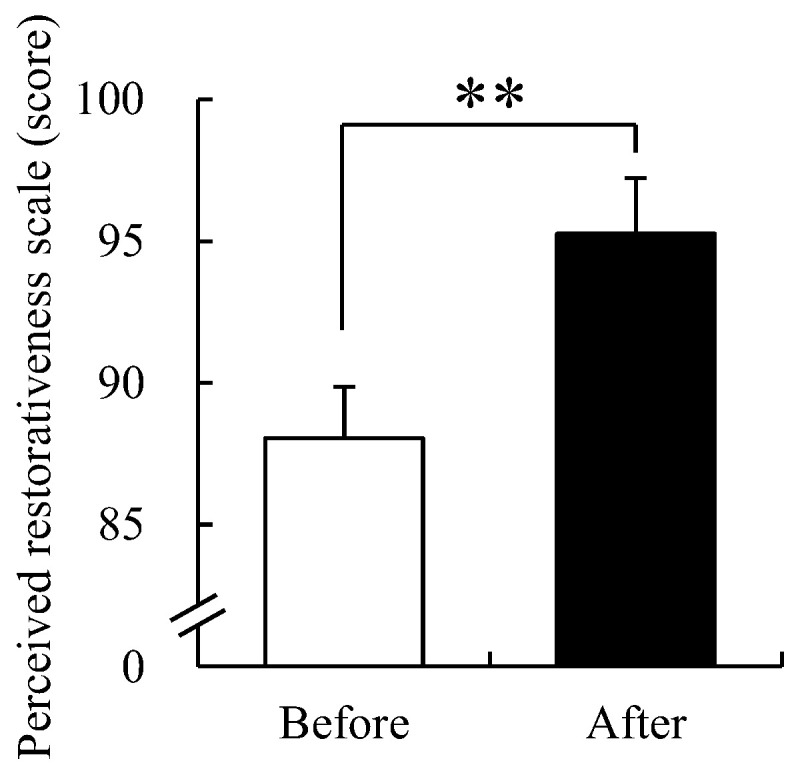
Changes in scores of the perceived restorativeness scale before and after the FCA. Data are presented as mean ± standard error (*n* = 61). Significance was verified by Wilcoxon signed-rank test; ** *p* < 0.01.

The perception of the recovery environment before and after participating in FCA was investigated using the PRS (Figure 9). The resulting average score of the perception of the recovery environment before participating in FCA was 89.91 ± 1.47 points, and the average score of the perception of the recovery environment after participating in FCA was 98.49 ± 1.68. This was a significant increase of 10.74% as compared prior to participation (*p* < 0.01). These results suggest that FCAs were effective in improving the perception of the recovery environment.

## 4. Discussion

The FCAs conducted in this study showed that the average working posture was at levels 1 and 2 as per OWAS classification. This indicated that the burden on the musculoskeletal system was low, and through calculation of the work intensity index using heart rate, all FCAs were found to be light-intensity activities. The results of this study differed from forest operations as labor, which is well known for its heavy high-intensity workload on the musculoskeletal system [37,38,39]. We believe that the reason for this is that through this study, we did not aim to maximize labor output or reach a target, but conduct a physical exercise-centered activity to promote health. In this study, a target work amount was not set for the participants and they were encouraged to rest if they felt fatigued during the activities, thus preventing the participants from straining their bodies.

Through this study, FCA participation was found to improve the subjective quality of life of the participants. FCAs offer a way to actively promote physical activity in forests, and the results of this study supported previous studies reporting the positive effects on the quality of life that could be obtained through similar physical activity [40]. FCAs were confirmed to enhance positive emotions while reducing negative emotions in participants, which is consistent with results of previous studies, which showed that experiencing nature can improve emotional wellbeing [41].

Additionally, FCAs were found to increase self-esteem. This is consistent with previous studies, which showed that self-esteem increased in individuals participating in “green exercises” such as walking, cycling, and environmental conservation activities [42]. The results of this study support those from previous studies stating that contact with the forest is effective in enhancing self-esteem. Additionally, a recovery environment refers to an environment where one can recover from mental fatigue, and the natural environment has been reported to be the best recovery environment [43]. This study found that FCAs were effective in improving the perception of the recovery environment, and is in support of the Attention Restoration Theory [43].

To summarize these results, the FCA program in which citizens participated, can provide positive effects on a physical and psychological level as a light-intensity activity that does not burden the musculoskeletal system. However, it is important to distinguish between forestry work performed by citizens and that performed by foresters. Forestry is one of the most demanding and dangerous jobs in numerous countries, and forestry workers have been reported to suffer from negative health effects due to long working hours, lack of rest, and high work stress [44]. In contrast, this study conducted forestry activities in a setting where citizens participated under the following circumstances: “do not set the workload”, “select the type of forestry activity suitable for one’s physical condition and preference”, and “take a break whenever you feel fatigued during the activity”. Under these circumstances, we hope that forestry activities in which citizens participate with the purpose of forest conservation will be recognized as a type of health-promoting physical activity.

The results of this study are based on the Biophilia Hypothesis that suggests that nature has a positive effect on human health [45] and strongly supports the psycho-evolution theory [46]. It is also consistent with numerous previous studies that investigated the effects of forestry activities on the emotional recovery and wellbeing of the human body. In particular, in the case of the United Kingdom’s Green Gyms, a program in which residents participate in local ecosystem management projects, it has been confirmed that participation enhanced life satisfaction, improved health, and increased self-confidence [47,48]. Uehara and Itoh (1999) reported that long-term forest work performed over a period of 1 year improved the physical ability, communication skills, emotional stability, and daily life activities of disabled individuals [49]. These results are consistent with our findings, and the purpose of the study was also quite similar.

The present study has limitations. First, the age of the study participants is biased toward those in their 20s. However, we believe that this study shows that FCAs can provide an opportunity to increase the physical activity participation rate of the generation in their 20s. In addition, FCA participation improves physical and psychological conditions, suggesting their potential use as exercise or occupational therapies; this warrants follow-up studies to scientifically elucidate detailed measurements and effects of FCAs on range of motion and muscle activity. Second, this experiment was conducted with a single group and lacked a control group. Thus, we believe that additional studies, including a comparison with a control group, are necessary. Third, FCAs were conducted in short 20-min sessions with the study participants participating only once. Therefore, further studies with longer FCA sessions are warranted for effective monitoring. Finally, if the researcher informs the subject about the purpose of the study before they participate in the study, some interview bias may occur when answering the post-questionnaire. Therefore, this aspect should be considered to avoid the occurrence of such bias.

Through this study, it is expected that the effectiveness of forestry education can be improved by providing motivation to raise awareness of the importance of sustainable forestry activities and the necessity of FCAs.

## 5. Conclusions

This study was conducted to investigate the effects of FCA participation on the physical and mental health of participants, and two conclusions were drawn as follows: First, FCAs, including pruning, stacking cut branches, and vine-removal, are light-intensity physical activities with a mild burden on the musculoskeletal system. Second, empirical evidence was presented that FCAs improve mental health and subjective quality of life, and induce mental recovery. To summarize, FCAs allow the maintenance of an environmentally healthy and sustainable forest ecosystem while having a beneficial effect on participants’ physical and psychological health, thus fostering the coexistence of humans and forests.

## Figures and Tables

**Table 1:** The characteristics of the experimental sites.

	Site 1	Site 2	Site 3	Site 4
Forest type	Conifers	Conifers	Non-stocked forest land ^1^	Non-stocked forest land
Tree species	*Metasequoia glyptostroboides*	*Picea abies* (L.) H. Karst.	-	-
Type of FCA ^2^	Pruning Stacking the cut branches	Pruning Stacking the cut branches	Vine-removal	Vine-removal
Slope (°)	10	10	15	15
Height (m)	15	20		
DBH (cm)	16	14		

^1^ Forest land with 30% or less standing trees; ^2^ FCA: Forest Conservation Activity.

**Table 2:** OWAS action categories for evaluation of working postures.

OWAS Categories	Action Category Description
Action Category 1	□ Work postures usually have no particular harmful effects on the musculoskeletal system. □ No actions are needed to change work postures.
Action Category 2	□ Work postures have some harmful effects on the musculoskeletal system. □ Light stress, no immediate action is necessary, but changes should be considered in future planning.
Action Category 3	□ Work posture has distinctly harmful effects on the musculoskeletal system. □ The working method should be changed as soon as possible.
Action Category 4	□ Work posture with an extremely harmful effect on the musculoskeletal system. □ Immediate solutions should be found to change these postures.

**Table 3:** The classification of physical activity intensity by %HRR.

	%HRR					
	<20	20–39	40–59	60–84	≥85	100
PAI	Very light	Light	Moderate	Hard	Very hard	Maximal

%HRR: Percentage of heart rate reserve; PAI: Physical activity intensity.

**Table 4:** Percentage of working posture action categories in FCA.

FCA	Action Categories (%)					Average Action Category
	1	2	3	4	Total	
Pruning	58.75	26.25	2.50	12.50	100.00	1.69 ± 0.11
Stacking the cut branches	45.00	27.50	25.00	2.50	100.00	1.85 ± 0.14
Vine-removal	35.00	20.00	33.75	11.25	100.00	2.21 ± 0.12

FCA: forest conservation activity.

**Table 5:** The classification of PAI by %HRR.

FCA	HRmax	HRr	HRw	PAI
Pruning	197.8 ± 0.2	78.9 ± 1.5	119.4 ± 1.6	33.6 ± 1.6
Stacking the cut branches	197.7 ± 0.2	78.8 ± 1.7	118.6 ± 1.9	32.8 ± 1.8
Vine-removal	196.6 ± 0.3	81.9 ± 1.9	116.2 ± 2.6	29.9 ± 1.9

PAI: physical activity intensity; %HRR: percentage of heart rate reserve; FCA: forest conservation activity; HRmax: maximum heart rate; HRr: resting heart rate; HRw: working heart rate.

## Data Availability

The data presented in this study are available on request from the corresponding author.
